# Time Domain NMR Approach in the Chemical and Physical Characterization of Hazelnuts (*Corylus avellana* L.)

**DOI:** 10.3390/foods12101950

**Published:** 2023-05-11

**Authors:** Raffaella Gianferri, Fabio Sciubba, Alessandra Durazzo, Paolo Gabrielli, Ginevra Lombardi-Boccia, Francesca Giorgi, Antonello Santini, Petra Engel, Maria Enrica Di Cocco, Maurizio Delfini, Massimo Lucarini

**Affiliations:** 1 Department of Chemistry, “Sapienza” University of Rome, Piazzale Aldo Moro 5, 00185 Roma, Italy; 2 Department of Environmental Biology, “Sapienza” University of Rome, Piazzale Aldo Moro 5, 00185 Roma, Italy; 3 NMR-Based Metabolomics Laboratory (NMLab), “Sapienza” University of Rome, Piazzale Aldo Moro 5, 00185 Roma, Italy; 4 CREA-Research Centre for Food and Nutrition, Via Ardeatina 546, 00178 Rome, Italy; 5 Department of Pharmacy, University of Napoli Federico II, Via D. Montesano 49, 80131 Napoli, Italy; 6 Council for Research in Agriculture (CREA), Office for International and Institutional Cooperation, Via Archimede 59, 00197 Rome, Italy

**Keywords:** Tonda Gentile Romana, Tonda di Giffoni, hazelnut, NMR relaxometry

## Abstract

‘Tonda Gentile Romana’ and ‘Tonda di Giffoni’ (*Corylus avellana* L.) are two Italian hazelnut cultivars, recognized under the quality labels “Protected Designation of Origin” (PDO) and “Protected Geographical Indication” (PGI), respectively. Hazelnut seeds are characterized by a complex microstructure and the presence of different physical compartments. This peculiarity has been studied and evidenced by Time Domain (TD) Nuclear Magnetic Resonance (NMR) experiments. This technique allowed the assessment of the presence of different diffusion compartments, or domains, by evaluating the distribution of the spin–spin relaxation time (T_2_).The aim of this research was to develop a method based on ^1^H NMR relaxometry to study the mobility in fresh hazelnut seeds (‘Tonda di Giffoni’ and ‘Tonda Gentile Romana’), in order to determine differences in seed structure and matrix mobility between the two cultivars. TD-NMR measurements were performed from 8 to 55 °C in order to mimic post-harvest processing as well the microscopic textural properties of hazelnut. The Carr–Purcell–Meiboom–Gill (CPMG) experiments showed five components for ‘Tonda Gentile Romana’ and four components for ‘Tonda di Giffoni’ relaxation times. The two slowest components of relaxation (T_2,a_ about 30–40% of the NMR signal, and T_2,b_ about 50% of the NMR signal) were attributed to the protons of the lipid molecules organized in the organelles (oleosomes), both for the ‘Tonda Gentile Romana’ and for the ‘Tonda di Giffoni’ samples. The component of relaxation T_2,c_ was assigned to cytoplasmic water molecules, and showed a T_2_ value dominated by diffusive exchange with a reduced value compared to that of pure water at the same temperature. This can be attributed to the water molecules affected by the relaxation effect of the cell walls. The experiments carried out as a function of temperature showed, for ‘Tonda Gentile Romana’, an unexpected trend between 30 and 45 °C, indicating a phase transition in its oil component. This study provides information that could be used to strengthen the specifications underlying the definitions of “Protected Designation of Origin” (PDO) and “Protected Geographical Indication” (PGI).

## 1. Introduction

The scientific name of hazelnut is *Corylus avellana*, a fruit tree belonging to the *Corylus* genus within the *Betulaceae* family. In botanical terms, the fruit is a diclesium (seed and shell), whose woody pericarp contains a sweet and oily seed (hazelnut), which is consumed cooked or raw.

Turkey is the main hazelnut producing country (up to 70% of the world total production), followed by Italy (15–20%), USA (5%) and other countries [1]. In Italy, hazelnut production is mainly concentrated in four regions: Latium ranks first with 56,000 tons produced in 2018, followed by Campania with 42,000 tons, Piedmont with 32,000 tons, and Sicily with 12,000 tons [2].

Some Italian hazelnut varieties are currently recognized under the PGI, (i.e., ‘Tonda Gentile delle Langhe’ and ‘Tonda Gentile Romana’ varieties) or under the PDO, i.e., ‘Tonda di Giffoni’ variety [3].

Hazelnuts are well known and consumed all over the world. Their peculiar nutritional and sensory properties make them appreciated not only as a fruit but also as ingredients in “picnic creams”, sauces (i.e., the “romesco” sauce in Spain), snacks, sweets (cakes, pastries, biscuits, nougats, etc.) and ice creams.

The chemical composition of hazelnuts is quite variable and it is strongly influenced by both pedoclimatic conditions of the growing site and the cultivar; Król and Gantner [4], showed, in hazelnut samples from different origins, high variability in the oil (ranging from 50.81% to 67.45%), protein (ranging from 7.03% to 22.06%) and moisture (ranging from 2.70% to 5.6%) contents with the highest oil concentration observed in countries with a warm climate (e.g., Spain and Italy). Muller et al. [5] found, in a study with 15 hazelnut varieties, that each hazelnut cultivar has a specific nutrient profile: the oil content ranging from 50.3% to 64.8%, the protein content ranging from 10.2% to 22.1% and the moisture content ranging from 3.3% to 4.5%.

Nonetheless, the common knowledge of hazelnuts being rich in fats has limited their consumption for a long time. However, only a few decades ago nutrition guidelines for people suffering from cardiovascular diseases recommended a reduced-fat diet, and hence nut consumption, in this case, was not recommended by nutritionists [6]. However, nuts are an important part of the Mediterranean diet, which is now recognized as a healthy diet considering that mortality rates linked to cardiovascular diseases are quite low in Mediterranean populations [7]. Over the last decade, much research has been carried out, focusing on potential health effects related to the consumption of nuts. Epidemiological studies have associated the frequency of nut intake with the reduction in the risk associated with some chronic diseases, such as cardiovascular diseases and even certain types of cancers [8,9,10]. For this reason, several years ago the Food and Drug Administration (FDA) of the USA approved a health claim for many nuts (almonds, hazelnuts, peanuts, pecans, some pine nuts, pistachio nuts, and walnuts) highlighting the positive effect of nut consumption on some pathologies if part of a healthy and balanced diet [11].

The abovementioned scientific evidence is associated with the chemical composition of nuts, in particular to the fat fraction, in which there are numerous compounds capable of carrying out a protective action for human health. In fact, among all nutrients, fats are predominant in hazelnuts, with contents ranging from 50% to over 70%.

In hazelnuts, an average fat content of 62–65% ensures a harmonious expression of aroma, scent, and texture; higher fat quantities are the prerequisite for faster rancidity and deterioration of the product [12].

The lipid fraction is mainly composed of oleic acid, followed by linoleic, palmitic, and stearic acids. These four fatty acids together represent more than 95% of the total hazelnut lipids [13,14,15,16].

Hazelnuts, in addition to their high fat content, are a good source of proteins, carbohydrates, fiber, minerals, vitamins (such as vitamin E), phytosterols, and phenolic antioxidants [17,18,19].

Although hazelnuts are rich in fat, recent data suggest that the inclusion of hazelnuts into the diet indicates a trend toward the reduction of total cholesterol, without decreasing high-density lipoprotein cholesterol and not adversely affecting body weight and its composition [20,21].

One of the hypotheses provided to justify this evidence is that, in comparison, the energy absorption from walnuts is incomplete, probably due to the structure of the granules where the fat is stored in the kernel, which allows only a partial release of fatty acids during digestion [22]. Indeed, lipids in oleaginous seeds are stored in oil bodies (OB), which are also known as oleosomes, which have a diameter of up to a few micrometers and are abundant in seeds and nuts [23]. Oleosomes are organelles formed by a core of neutral lipids, mainly triacylglycerols, surrounded by a layer of a unique protein-phospholipid membrane. In plants, oleosomes can be expressed in different organs and tissues and may change in response to specific environmental conditions [24].

NMR is undoubtedly the most suitable technique for studying the dynamic state of liquid components in heterogeneous systems and TD ^1^H NMR is widely used for food products’ characterization. The inter- and intra-molecular interactions modulated by molecular dynamics can be efficiently studied with NMR relaxometry. The technique has already proven to be helpful to investigate changes occurring in food products due to different processing techniques and to detect food shelf-life, etc. [25,26]. TD ^1^H NMR spectroscopy has been successfully used to study molecular mobility in numerous food systems to reveal matrix changes occurring during processing and/or storage of different hazelnuts [27]. In this way, it was possible to characterize foods by determining both the decay rate and the amplitude of the ^1^H NMR signal. The T_2_ relaxation curve in heterogeneous systems is represented by a multiexponential decay curve that can be attributed to the presence in the sample of elements with structural differences [28,29].

Correct interpretation of multiexponential curves in heterogeneous food systems relaxation is difficult but is a precondition fundamental to revealing how the relaxation times are affected by the system morphology. A diffusive and chemical exchange model suggested by Hills and co-workers [30] proposed that water molecules diffuse to the biopolymer surface and exchange with biopolymer protons, leading to enhanced surface relaxation [25,30]. By the diffusive and chemical exchange model application, multiexponential water and oil components in hazelnuts have been identified. The single relaxation components do not indicate a different site but reflect the diffusion of the water molecules between different sites and oil molecules in the organelles, their morphology, water–biopolymer interactions and the seed texture. Several studies were carried out in order to evaluate the quality of different nut species, such as the macadamia kernel [31], Brazil nuts [32]. Other studies were carried out applying the ^1^H TD NMR technique to detect the quality of inshell hazelnuts [33] and to classify hazelnut oil [34], but to the authors’ knowledge no paper went into further detail related to the chemical–physical characterization of hazelnuts. The aim of this research was to develop a TD ^1^H NMR based method to study the matrix mobility in fresh seeds of two hazelnut varieties (‘Tonda Gentile Romana’ PDO and ‘Tonda di Giffoni’ PGI) and explore if NMR can distinguish the differences between the two cultivars in structure and matrix mobility.

## 2. Materials and Methods

### 2.1. Origin of the Samples

Samples of cultivars ‘Tonda Gentile Romana’ and ‘Tonda di Giffoni’ were taken from the hazelnut field collection of ARSIAL (Regional Agency for Innovation and Development of Agriculture in Latium, Italy), located in a typical hazelnut-growing area of the Viterbo province, at “Le Cese” (coordinates 42.348229; 12.194091) [35].

The orchard from which samples were taken is established on a sandy clay loam soil with pH 6.1, 2.2% organic matter and a low total calcium content (7.4 g kg^−1^).

Each cultivar is represented by three multi-stemmed plants growing at spacings of 4 × 5 m and trained as open vase, irrigated through a sub-irrigation system and managed with standard orchard management techniques and an integrated pest management plan.

Fertilization provides an annual distribution of 80 units of nitrogen (N), 50 units of potassium (K_2_O) and 100 units of phosphorus (P_2_O_5_) [36].

### 2.2. Sampling

‘Tonda di Giffoni’: the nut is dark brown, with a roundness index = 1.0, average size 19.0 × 20.7 × 18.2 mm, average weight 2,5 g, thin shell; average seed size 14 × 13.7 × 11.5 mm, average weight 1.16 g, kernel/nut ratio 46%, high blanching rate. Harvest date is the end of August.

‘Tonda Gentile Romana’: the nut is dark brown, roundness index = 1.0, average size 19.6 × 18.6 × 18.2 mm, average weight 2.5 g, thin shell thickness, average seed size 14.2 × 12.8 × 13.3 mm, average weight 1.16 g, high blanching rate. Harvest time is in mid-September.

Fruits were collected at peak harvesting time, i.e., when 50% of fruits have fallen off the plants. For both cultivars, 1 kg of product (about 400 fruits) were selected, based on quality criteria for fresh produce (i.e., intact, sound and clean shells). After harvest, the whole hazelnuts, having a humidity of 14–15%, were placed in open paper bags and kept in a dry place at room temperature for 15–20 days, until they reached a humidity of 12% of the whole nut and 6% of the seed (in accordance with [37]).

No significant differences were observed between the cultivars regarding the moisture content, neither for whole nuts nor seeds.

Afterwards, they were stored at 4 °C until the beginning of the trial a few days later. Sampling then consisted of the elimination of deformed, flat, wrinkled, dry and untypically colored seeds, as well as those damaged by biotic agents (insects, fungi). A further requirement for the choice of seeds was the absence of rancidity, atypical smell or taste.

### 2.3. TD NMR Spectroscopy

The TD NMR experiments were performed on a Minispec mq 20 pulsed NMR spectrometer (Bruker, Milano, Italy) with an operating frequency of 20 MHz for proton (magnetic field strength of 0.47 T). The NMR spectrometer was equipped with an external thermostat, Julabo F25 (Julabo Labortechnik GmbH, Germany) to maintain the desired temperature conditions.

To investigate the texture of hazelnuts and get information on the molecular mobility of water and oil in them, NMR measurements were carried out at different temperatures in the range from 8 °C to 55 °C, choosing each temperature value based on the trend of the measurements of the cultivar one (for this reason the temperatures do not coincide in the two cultivars’ measurements).

In fact, the macroscopic textural properties of the sample are related to proton mobility on a molecular scale.

Furthermore, the T_2_ values of the lipid components are particularly sensitive to the increase in temperature due to their structure: the lipid chains (both in the bilayer, both in the micelle) increase their mobility passing from a solid state (gel) to a liquid one, in which lipid molecules can diffuse much more freely. This behavior allowed us to uniquely assign the components of relaxation.

Finally, the water NMR relaxation times have a dependence on the temperature because they are related to molecular motions (conveniently described in terms of correlation times). The increase in temperature increases the motion of molecules and, therefore, the correlation times. When water molecules are in different diffusive domains, correlation times increase with increasing temperature in different ways, related to different effects of the network structure motions and chemical exchange rates in each domain.

Before each measurement, samples were allowed to equilibrate to the measuring temperature for at least 20 min, inside the probe, after having left the samples in the thermostat at the same temperature. Analyzed samples were subjected to a temperature ramp.

Samples were prepared by selecting a set of hazelnuts for each cultivar, homogeneous in size (determined by the maximum diameter of the normal section) with kernels that appeared intact (slight surface imperfections were not considered a defect), healthy (any seeds affected by rot were excluded) and free from visible external contaminants, such as insects, parasites or mold filaments. The kernels were split along the hypocotyl axis between the two cotyledons, and pieces of seed with a normal section size higher than 9 mm were excluded.

Subsequently, the selected samples (half kernels, whole) were introduced into an NMR tube, with an external diameter of 10 mm, without any additional treatment. Four samples from four different hazelnuts were prepared for each variety. Then, each tube was sealed with a Teflon insert to prevent evaporation.

All NMR measurements were performed using the same relaxation delay, overestimated with respect to the minimum value (five times T_1_) to ensure the complete relaxation of the NMR signal and the sampling reproducibility of the measurements (same sampling conditions).

For each sample and each temperature, the goodness of the RD was verified through a preliminary measurement of T_1_ by means of an inversion reset sequence.

#### 2.3.1. Analysis of the Liquid Content (X)

The liquid phase (referred to water and lipid molecules) content, X, was determined by analyzing the Free Induction Decay (FID) signal after 90° RF pulses of 2.9 μs. The measurement was repeated 160 times with a relaxation delay of 1 s (RD) between two following scans, in order to assure complete relaxation of the FID signal. RD was determined using the following relation (1):

(RD − 90°_x_ − acq)_n_ (1)

The interpolation of the experimental curves of the FID was achieved through a non-linear based fitting on the least squares method using the Marquardt algorithm, implemented in SigmaPlot scientific software (version 9, Systat Software Inc., San Jose, CA, USA).

The FID due to the protein and water protons network and water and lipid protons envelope was analyzed by applying two relaxation processes: a Gaussian-type relaxation process related to ‘solid’ polymer protons and a Lorentzian relaxation process related to ‘liquid phase’ protons according to the *expfit* algorithm [38] carried out in MatLab version R2009a (The Mathworks Inc., Natick, MA, USA).

#### 2.3.2. Spin Eco Sequence: Simultaneous Determination of Moisture and Oil Content

Simultaneous determination of moisture and fat content via TD NMR by Hahn’s Spin Echo sequence (SE) is a standard International Method [39,40,41].

#### 2.3.3. CPMG Sequence: Determination of Spin–Spin Relaxation Times

Transverse relaxation times (T_2_) were assessed by the CPMG pulse sequence [42] with a pulse spacing, τ_cp_, between two following 180° pulses, of 0.08 ms; 400 data points of the relaxation curve were acquired after each echo in order to detect the shortest T_2_ values, while the other 3000 data points were acquired after each 5th echo in order to detect the longer T_2_ value samples, as an average of 64 repetitions, and with a RD of 1.5 s to assure complete relaxation of the nuclear spins between repetitions (ca. 5–10 T_1_).

The transverse magnetization decay curves were analyzed using distributed exponential fitting analysis according to the regularized inverse Laplace transform, based on the CONTIN program developed by Provencher [43,44] to determine the number of relaxation components, because it does not require any assumption of their number. The program was carried out in MatLab [45] using rilt scripts [46] with logarithmically distributed relaxation times from 0.01 ms to 10,000 s.

According to the inverse Laplace transform results, a model was defined to fit the experimental CPMG, data with a discrete method based on a sum of exponential, with an echo amplitude at the time τ A(τ), spin–spin relaxation time and amplitude of i-component T_2,i_ and Ai, respectively, and a constant to represent the decay curve noise.

Multi-exponential regressions were performed by the Levenberg–Marquadt method [47] for non-linear regression, which followed a least-squares minimization strategy and was carried out in SigmaPlot version 9 [48] using an in-house script. Correlation coefficients ® and the least-squares test was used to determine the goodness-of-fit of the data, and an F-test on the reduced least-squares ratio was used to determine whether one exponential number for a given set of data was better than an alternative number. This method cannot determine with sufficient accuracy components characterized by T_2_ values of the same order of magnitude.

#### 2.3.4. Cryo-SEM of Hazelnut Section

SEM images: The hazelnut samples for SEM images were cryo-fractured by transfer out under liquid nitrogen. Cryo-fractured samples were freeze-dried and mounted on an aluminum stab using double-sided carbon tape. The sample was coated with a 10 nm thick gold film using a sputter coater. Coated samples were examined using an electron acceleration voltage of 20 keV, in both the secondary and the backscattered electron modes using a LEO 1450 VP.

#### 2.3.5. Statistical Analysis

Results were expressed as the mean ± SD of at least four independent experiments. The statistical significance was assessed by one-way analysis of variance (ANOVA) followed by Bonferroni’s test using SPSS 19.0. Differences were considered statistically significant when *p* < 0.05

## 3. Results and Discussion

Oil-based agro-food products, such as hazelnut seeds, are complex heterogeneous systems in which the few water molecules present in the matrix interact with proteins, triglycerides and the polar heads of phospholipids [49]), determining the structural organization of lipid molecules in micelles and lipid bilayers, and therefore also their mobility. Depending on the chemical composition of the system, the water molecules and lipids are affected by different physical (electrostatic, hydrophobic, etc.) and chemical interactions (chemical exchange) according to the structural organization such as micelles, double layers, etc. [27]. This determines different physical “compartments”, specifically diffusive domains, characterized by a different mobility of the lipid chains through which the water molecules can move (diffusive exchange), hence experiencing different magnetic field environments [27]. The dynamic NMR parameters (longitudinal and transversal relaxation times) are very sensitive to the motion state of molecules, and their measurement and analysis significantly contribute to the understanding of the system subject to this study.

### 3.1. Measurements of the Liquid Phase Content, X, as a Function of Temperature

The liquid phase content X, of seeds of hazelnut cultivars ‘Tonda Gentile Romana’ and ‘Tonda di Giffoni’ were determined by analysis of the FID signal.

**Figure 1:**
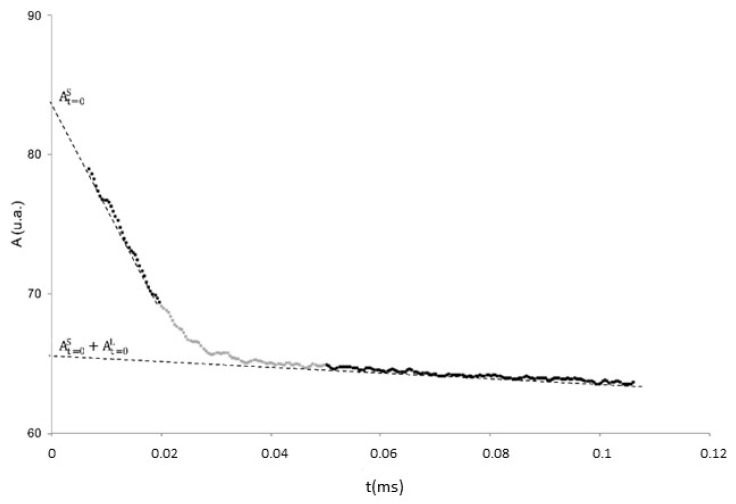
FID of a sample of seeds of hazelnut cultivar ‘Tonda Gentile Romana’, at a temperature of 15 °C. Data points used for the determination of total proton contents are given in black (straight line extrapolation in dotted line) and in the liquid phase (line of extrapolation in dashed line).

Quantitative analysis of the FID decay was used to determine the content X-percentages of the liquid component (oil and water components) with respect to the total, liquid and solid (all protons, of protein networks, of crystallization water, etc., whose relaxation time is of the order of microseconds) (X%), following the relation indicated below:

$$X\%=\frac{A_{t=0}^{L}}{A_{t=0}^{L}+A_{t=0}^{S}}A_{t=0}^{L}/\left(A_{t=0}^{L}+A_{t=0}^{S}\right)\;$$

where $A_{t=0}^{L}$ and $A_{t=0}^{S}$ are the amplitudes of the NMR signal at zero time (immediately after the 90 °rf pulse), which are directly correlated to the relative abundance of hydrogen atoms, due, respectively, to the protons of the “liquid” phase and all protons present in the sample, both in the “solid” and in the “liquid” phase, as shown in Figure 1 for a sample of ‘Tonda Gentile Romana’ at 25 °C.

**Figure 2:**
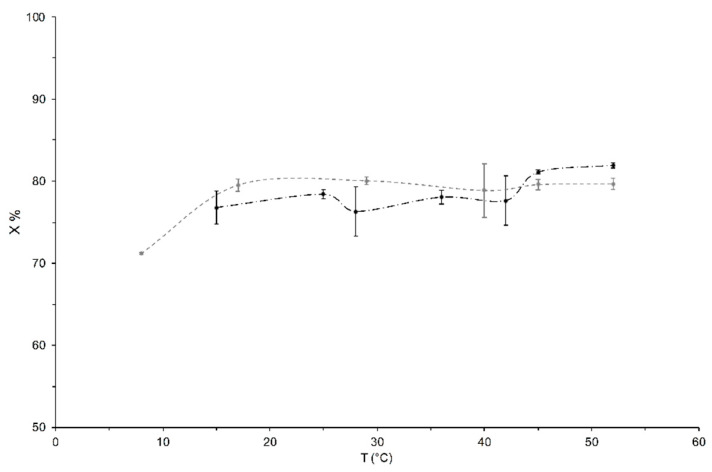
Liquid content X% versus temperature for the hazelnut samples of ‘Tonda Gentile Romana’ (black dots) and ‘Tonda di Giffoni’ (grey points). Values are an average of four independent measurements with the relative standard deviation.

The liquid phase percentages (X) for ‘Tonda Gentile Romana’ and ‘Tonda di Giffoni’ were measured at different temperatures (from 8 °C to 55 °C) and are summarized in Figure 2 as an average of four independent measurements.

The analysis of total fat and moisture by TD-NMR is a traditional standard method applied to samples whose moisture content does not exceed a certain limit, i.e., moisture should be present in the sample as bound water.

The minispec standard method assumes that all fat protons exhibit an NMR relaxation time longer than that of the bound water.

It is noteworthy that the X% is directly proportional to the amount of oil in hazelnut, which is one of the main factors in defining hazelnut quality. In fact, oil is the basic constituent of the aroma and flavor of hazelnuts and of its nutritional value, but it is also the factor that mainly affects the shelf-life of the seeds.

The behavior of ‘Tonda Gentile Romana’ vs. temperature shows a discontinuity at around 30 °C and 45 °C, which indicates a phase transition related to its liquid component. This transition is not visible in the sample of ‘Tonda di Giffoni’ in the temperature range considered, which, however, shows a significant increase in the value of X between 8 and 15 °C.

### 3.2. Measurements of the Spin–Spin Relaxation Times

Proton relaxation is affected by variations in mobility and exchange phenomena to which they are subjected, thus reflecting in heterogeneous systems the conditions of exchange and different mobility of nuclear spins [50]. This means that, in the agri-food sector, the T_2_ measurements reflect the chemical composition, the nature and the structure of food [25,51].

The main experimental evidence is an increase in the rate of proton relaxation of water molecules (generally, one or two orders of magnitude), as well as a different trend of relaxation times, T_2,oss_, compared to the linear one of pure water which is dominated by the dipole–dipole interactions.

Therefore, the analysis of the proton NMR relaxation curves is a potential tool to provide information on the distribution of lipid and water molecules as well on the morphology and dimensions of the lipid diffusive domains in the lattice of hazelnut seeds.

**Figure 3:**
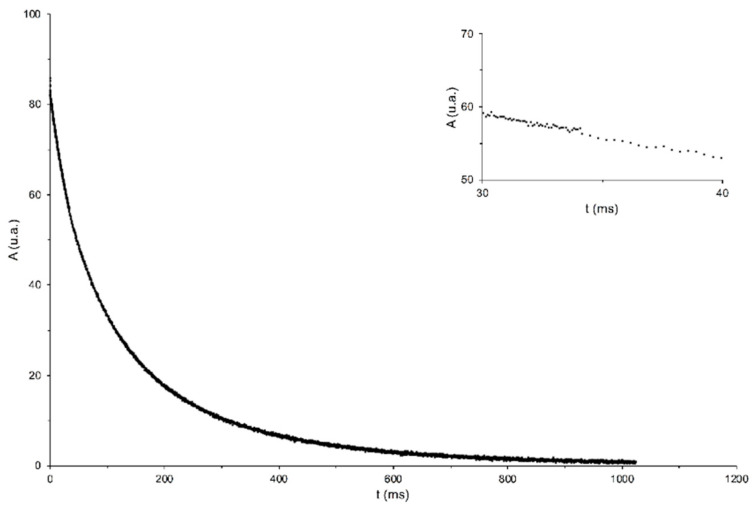
Transverse magnetization decay curves (echo signal vs. echo time) obtained from a CPMG experiment for hazelnut seed samples of cv ‘Tonda Gentile Romana’ at a temperature of 15 °C.

The curves of transverse relaxation were measured by CPMG Sequence. Figure 3 shows a typical experimental decay curve of transverse magnetization obtained for the ‘Tonda di Giffoni’ sample at a temperature of 15 °C.

**Figure 4:**
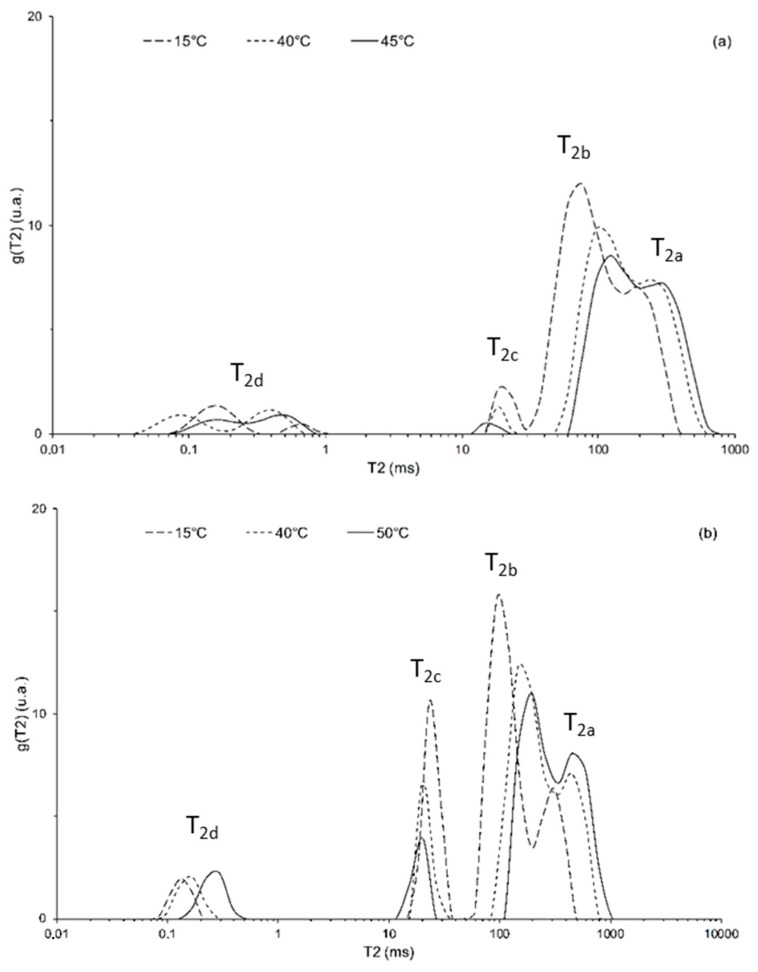
Distribution of transverse relaxation times (normalized amplitudes with respect to the total area of the distribution vs. logarithmically distributed relaxation times) from an inverse Laplace Transform of the CPMG experimental curves at three different temperatures for (**a**) ‘Tonda Gentile Romana’ and (**b**) ‘Tonda di Giffoni’ hazelnuts.

The CPMG curves of the protons of water and lipids in all samples were found, as normally occurs in heterogeneous systems, as multi-exponential, characterized by the presence of five components for ‘Tonda Gentile Romana’ (Figure 4a) and four components for ‘Tonda di Giffoni’ (Figure 4b); for ‘Tonda Gentile Romana’, the two faster components found (Figure 4a) were not resolved and in this order we have indicated them as a single component resulting from an average of them (Table 1).

Figure 4 shows, as an example, the distribution of spin–spin relaxation times obtained by the inverse Laplace transform of the experimental curves CPMG seed samples of hazelnut cultivars: (a) ‘Tonda Gentile Romana’ and (b) ‘Tonda di Giffoni’ at three different temperatures.

The analysis of NMR proton relaxation curves can provide information on the distribution of lipid and water molecules in the hazelnut seed lattice and on the morphology and dimensions of the diffusive domains of lipids inside the seed. As can be seen in Figure 4, the analyzed seeds are characterized by a complex microstructure with the presence of several physical compartments. As already mentioned, “compartments”, or different “diffusive domains”, are recognized in the distribution of spin–spin relaxation times, as well-defined peaks in the T_2_ relaxation times. For both hazelnut cultivars, the T_2_ relaxation was characterized by a minor population with a relaxation time of a few ms and a major population with a relaxation time around 70–1000 ms. The position of the major component clearly shifted towards higher relaxation times with increasing temperature.

Figure 4 also highlights a first difference between the two cultivars, a greater resolution of the peaks in the distribution of the ‘Tonda di Giffoni’ cultivar compared to that of the ‘Tonda Gentile Romana’ cultivar. It is, therefore, possible to hypothesize that these two components of the relaxation time represent different oleosomes whose dimensions are distributed over two relatively broad ranges of the relaxation times centered on the maximum of peaks.

The transverse relaxation curves were analyzed by the inverse Laplace transform for signal analysis of the experimental data to identify the distribution of transverse relaxation times (number of relaxation components and their amplitude) in each sample [43].

The values of the relaxation times of individual components (T_2,a_, T_2,b_, T_2,c_, T_2,d_) and their NMR signal percentage were obtained by discrete deconvolution.

Table 1 shows the values of transverse relaxation times, and the percentage of the NMR signal, averaged over four samples, for the seeds of ‘Tonda Gentile Romana’ and ‘Tonda di Giffoni’, obtained by deconvolution of the discrete NMR experimental data.

In the present work, the assignment of the various components of the relaxation to different physical parts of the system was carried out based on knowledge of the structure of the system, and by analyzing the values of the different relaxation times and the relative percentages of the NMR signal. The assignment of the relaxation components, characterized by different intrinsic T_2_ relaxation times, to several components in hazelnuts is not so obvious and requires some reasoning/explanations.

The two slower components of relaxation, with longest T_2_ values T_2,a_ = 186 ± 3 ms and 270 ± 4 ms and T_2,b_ = 68 ± 1 ms and 98 ms ± 1 at a temperature of 15 °C for ‘Tonda Gentile Romana’ and ‘Tonda Giffoni’, respectively, have been assigned to the protons of the lipid molecules arranged in oleosomes organelles.

These components are attributed to lipids thanks to the significant shift of their peaks, with the increase in temperature, due to the increase in lipid mobility. In plants, and in particular in seeds, oleosomes represent the storage form of triglycerides; the sophisticated oleosome membrane is made of a continuous monolayer of phospholipids and hydrophobic proteins and shields the triglyceride core by giving them physical and chemical stability. They appear in the form of small drops, with the polar heads oriented toward the aqueous phase and the lipophilic tails towards the lumen; proteins, defined oleosines, are present on the surface of the oleosomes, designed through natural evolution including structural proteins (such as oleosins and caleosins), enzymes (i.e., sterol dehydrogenases or steroleosins and lipases) and minor proteins (i.e., aquaporins) [52] They prevent the fusion of the different oleosomes and the contact of the acyl chains of the triglycerides with the enzymes present in the cellular material [24,53,54], but in relation to their functional group are considered to possess biological functions in signal transduction related to the synthesis or degradation of oil bodies [53]. It is interesting to notice (Table 1) that the two components attributed to lipids (T_2a_ and T_2b_) significantly increase along with temperature for both cultivars studied. This indicates that the mobility of lipids increases due to the fact that oleosomes are changing their structure under the influence of temperature.

These two components are widely distributed in the system; peaks in the distribution of T_2_, obtained by the inverse Laplace transform of the experimental curves CPMG, are not completely resolved and show a partial overlapping: the overlapping is more marked for Tonda Gentile Romana (Figure 4). It can therefore be assumed that these two components of the relaxation represent different oleosomes whose dimensions are distributed over two relatively wide intervals centered on the maximum of the peaks.

The component that relaxes with intermediate speed T_2,c_ = 22.4 ± 0.7 ms and T_2,c_ = 23.8 ± 0.2 ms at a temperature of 15 °C and 17 °C, respectively, for ‘Tonda Gentile Romana’ and ‘Tonda di Giffoni’, respectively, can be assigned to the cytoplasmic water molecules which undergo a restricted diffusion. The T_2_ of this component is minor compared to that of pure water at the same temperature

(T_2_, pure water = 2 s) by interaction with the protein structures of oleosine and other relaxation contributions, due to geometrical factors of restricted diffusion domain [55]. Traoré et al. [56] note how the chemical shifts value forecasts a small contribution to the exchange process at 20 MHz.

These water molecules are likely to interact with the cellular structures by diffusion from the bulk to the interface walls, which act as a relaxation sink, while T_2_ values are controlled by diffusion. If relaxation on the surface is fast compared with diffusion to the surface-diffusion limited relaxation regime, the observed water transverse relaxation rate is independent of the effective surface relaxation strength and is given by equation:

$$\frac{1}{T_{2,obs}}=\frac{1}{T_{2,w}}+\frac{\pi^{2}D}{4a^{2}}$$

where *T_2,obs_* and *T_2,w_* are, respectively, the measured and the water protons intrinsic relaxation times; *D* is the water self-diffusion coefficient at the measure’s temperature and “*a*” is the size of cellular compartments bound by surface relaxation walls ‘2*a*’ apart.

**Figure 5:**
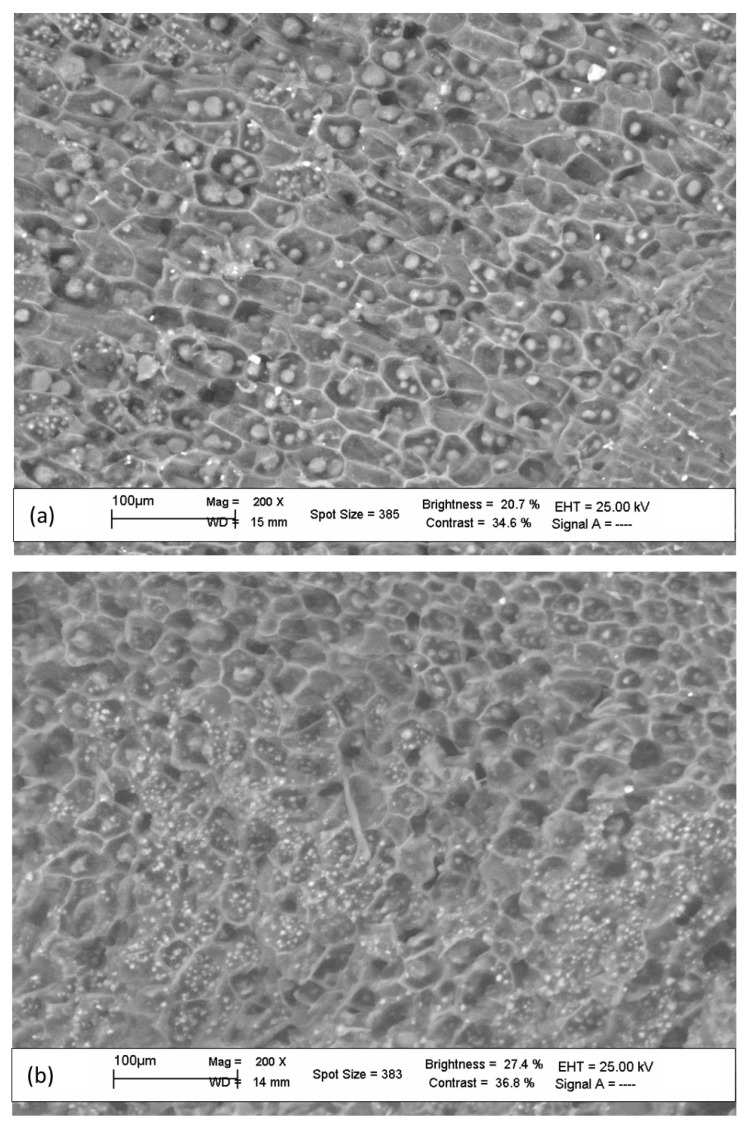
Cryo-SEM (scale bar: 100 μm) of hazelnut sections of (**a**) ‘Tonda Gentile Romana’ and (**b**) ‘Tonda di Giffoni’ cultivars. Oleosomes and cell structure walls are clearly visible.

Assuming at 30 °C that the T_2,c_ value is *T*_2*,obs*_, *T*_2*,w*_ = 2.88 ± 0.01, as measured by an independent experiment, and the value of D = 2.594 × 10^−9^ m^2^ s^−1^ [57], a maximum diffusive distance for water molecules in the cellular structures, of about 24 μm, was derived for the samples of both ‘Tonda Gentile Romana’ and ‘Tonda di Giffoni’. This order of magnitude is in good agreement with the average space for the compartments obtained from the SEM images reported in Figure 5. In these images, oleosomes of different sizes are also clearly visible.

The component that relaxes faster, T_2,d_ = 0.25 ± 0.01 ms and 0.14 ± 0.02 ms at 15 °C for ‘Tonda Gentile Romana’ and ‘Tonda di Giffoni’, respectively, can be attributed to the protons of the water molecules which are strongly immobilized within cell structures and whose relaxation time is significantly reduced compared to that of pure water at the same temperature [58].

This is due to the effect of the fast chemical exchange with the protons of the macromolecules of cellular structures. However, the discussion on chemical exchange processes is extremely complex [59] and goes beyond the scope of this research.

#### Measurements of the Relaxation Time Spin–Spin as a Function of Temperature

**Figure 6:**
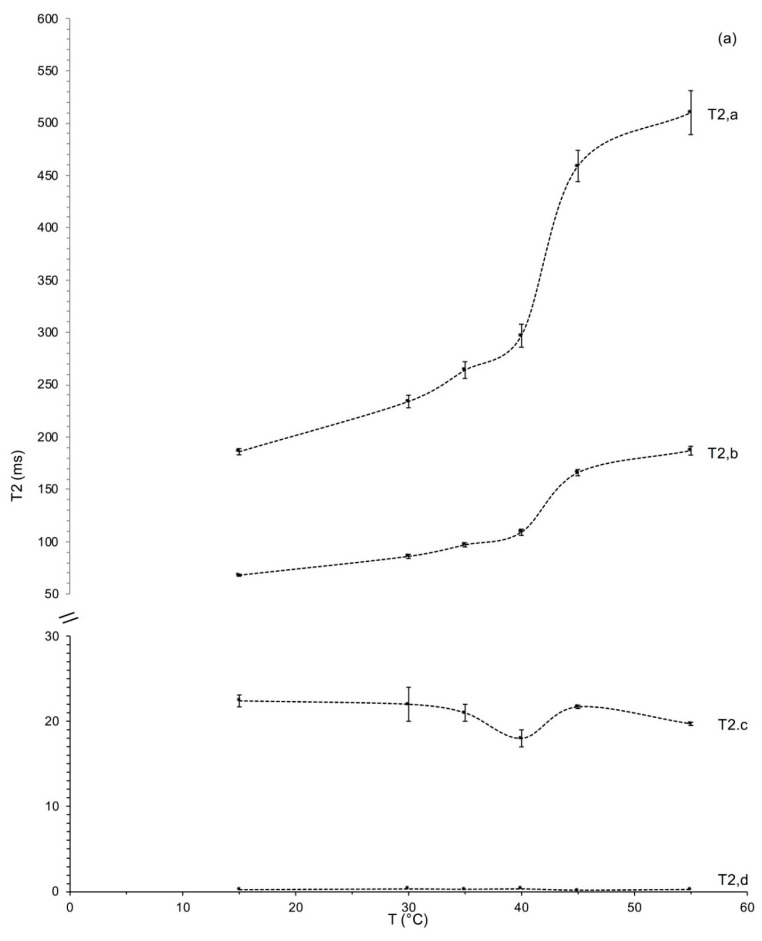

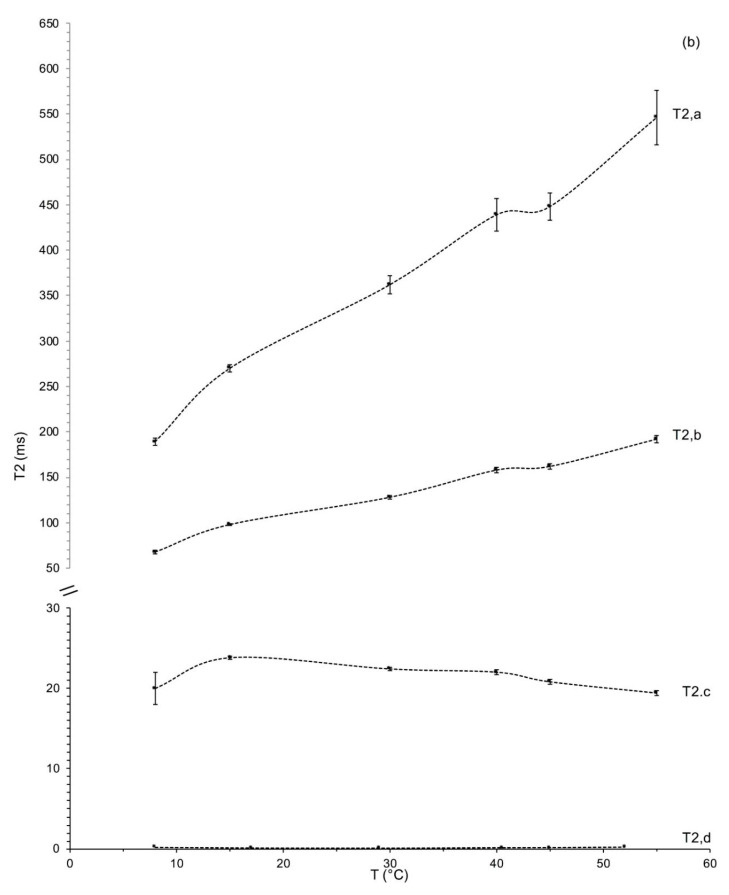
The different components of the transverse relaxation times of hazelnut samples of ‘Tonda Gentile Romana’ (**a**) and ‘Tonda di Giffoni’ (**b**) as a function of temperature. Values reported are an average of measurements of four samples and errors are the relative standard deviations.

Figure 6 shows the trend of spin–spin relaxation times as a function of temperature of the seed samples of ‘Tonda Gentile Romana’ (a) and ‘Tonda di Giffoni’ (b). The trend of T_2_ vs. T of ‘Tonda Gentile Romana’ (Figure 6a) shows an evident discontinuity between 35 °C and 45 °C for the lipid components, T_2,a_ and T_2,b_, and of the aqueous component T_2,c_ of relaxation. The trend presented by the lipid components T_2,a_ and T_2,b_, is typical of a lipid phase transition, therefore, linked to oleosomes. In fact, in literature it is well documented [60] that, with rising temperatures, the chains of fatty acids of triglycerides increase their mobility, thus inducing a change in the lipid physical state from the ordered gel phase, where the hydrocarbon chains are fully extended and closely packed (ordered gel-like structure), to the disordered liquid crystalline phase, where the hydrocarbon chains are randomly oriented and slightly packed. In the disordered state, however, fluid regions coexist with regions in the state of gel. Therefore, the analyzed ‘Tonda Gentile Romana’ seeds show a phase transition liquid crystal to fluid regions/gel state between 35 °C and 45 °C (shown as inflection points of the T_2_ vs. T curves of the components a and b in Figure 6a).

The trend presented by the aqueous component c is also typical of a sol–gel transition [55] as shown by the minimum point on the graph of T_2_ vs. T (Figure 6a).

**Figure 7:**
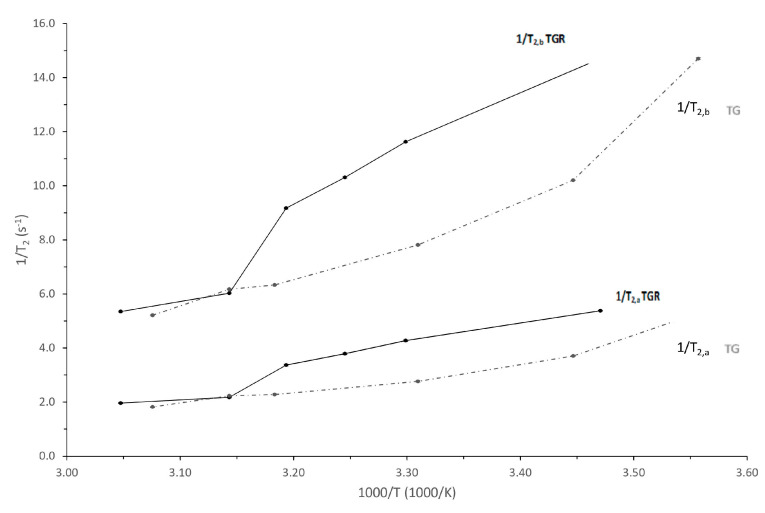
Arrhenius plot of the transverse relaxation rate (1/T_2_) of the two lipid components of hazelnut samples ‘Tonda Gentile Romana’ (TGR, black lines) and ‘Tonda di Giffoni’ (TG, grey lines) versus 1000/T. The values are the average and the relative standard deviations of measurements of four samples.

Contrarily, the T_2_ vs. T trend of ‘Tonda di Giffoni’ (Figure 6b) did not show such transition in the temperature range 35–50 °C. This different behavior could probably be attributed to the different composition of the proteins present on the membrane of the oleosomes present in the seeds of the two studied cultivars. In order to further verify the trends shown in Figure 6, the Arrhenius graph was elaborated, reporting the spin–spin relaxation rate (1/T_2_) vs. 1000/T, of the two lipid components, T_2,a_ and T_2,b_, in both cultivars (Figure 7).

From a careful analysis of the trends reported in Figure 6b and the Arrhenius graph in Figure 7, it is possible to hypothesize that this transition is shifted to a lower temperature (<15 °C). Indeed, in the Arrhenius graph the trends of the spin–spin relaxation rates vs. 1000/T of the two lipid components, a and b, of both cultivars show similar slopes in different temperature ranges which assume similar behaviors (that means similar energies of process activations) in different temperature ranges. The temperature criticality of this phase transition generally ranges between 10 and 40 °C (but can be higher), in relation to the fluidity of the membranes, which depends on the flexibility of the fatty acid chains and, therefore, on the degree of unsaturation of the fatty acids themselves, as well as on the length of their chains [60]. Hence, we hypothesize that the absence of transition in the temperature range analyzed for ‘Tonda di Giffoni’ could indicate that lipids in the seeds of these hazelnuts are more structured than in ‘Tonda Gentile Romana’. This confirms the results of the trend of the ratio X as a function of temperature (Figure 2) and the closer and more defined peaks in the distributions of relaxation times spin–spin (Figure 4) of ‘Tonda di Giffoni’ with respect to ‘Tonda Gentile Romana’.

## 4. Conclusions and Future Trends

Seeds of hazelnut cultivars ‘Tonda Gentile Romana’ and ‘Tonda di Giffoni’ were characterized by TD NMR. All samples showed a transversal relaxation (T_2_) characterized by the presence of five for TG Romana and four components for TD Giffoni. For TD Romana two of which are assigned to the lipids and the other three to the water molecules, while for TD Giffoni two are assigned to the lipids and the other two to the water molecules. Both systems are based on (i) the T_2_ relaxation time values, (ii) the relative percentages of NMR signal and (iii) the knowledge of the composition and the structure of the hazelnut seeds. The lipid components, T_2,a_ and T_2,b_, are attributable to the triglycerides constituting the oleosomes, i.e., the intracellular lipid structures characterizing the morphological structure of hazelnut seeds.

The presence of two lipid components characterized by different relaxation times for the two hazelnut cultivars is particularly interesting. These may be attributable to two lipid structures of different sizes, as also highlighted by the SEM images.

The two faster components of relaxation, T_2,c_ and T_2,d_ are, respectively, assigned to the protons of the cytoplasmic water molecules and to the protons of the water which is strongly immobilized within the cellular structures. From the relaxation time of water in the cytoplasm, characterized by a limited diffusion relaxation regime, it was possible to determine a maximum diffusive distance for water molecules in the cellular structures of about 24 μm for both analyzed cultivars.

The trends of T_2_ along with temperature, for ‘Tonda Gentile Romana’, showed an oleosomes phase transition from liquid crystal (ordered gel-like structure) to disordered or slightly packed acyl chains between 40 °C and 45 °C; as the temperature rises, the fatty acid chains of triglycerides increase their mobility causing an increase in the fluidity of the lipid membranes. At the critical temperature (between 40 and 45 °C) at which the lipid micelles change their state, the aqueous component c shows a sol–gel transition. ‘Tonda di Giffoni’, in the investigated temperature range (8–55 °C), did not show this transition. Finally, this study provides the theoretical framework necessary to employ TD-NMR based techniques to evidence possible damage of the seeds, which is one of the major concerns in hazelnut production. In particular, since the mobility of the lipids in the matrix is influenced by several factors such as the stiffness of the oleosome, its chemical composition as well as its oxidation state, the T_2_ times as well as their relative areas could be employed to monitor the shelf-life and ongoing ripening processes of hazelnuts and other nut crops. The study carried out gives important indications regarding the stability of hazelnut fats. The chemical–physical stability of fats during fruit storage is in fact an important parameter to establish product quality. These measurements make it possible to analyze the fruit without any treatments and can therefore give important indications on the quality of the fats which represent the fundamental parameter in determining hazelnut quality. Moreover, since this study was carried out taking into account the ripening stage, the production area and the processing of the nuts, it also provides a larger amount of information that could be used to strengthen the specifications underlying the definitions of “Protected Designation of Origin” (PDO) and “Protected Geographical Indication” (PGI) for the examined cultivars, and provide a useful reference for future similar studies.

## Figures and Tables

**Table 1:** Proton transverse relaxation time values (T_2,a_, T_2,b_, T_2,c_, T_2,d_) and signal percentages at different measuring temperatures (8–55 °C) in two hazelnut samples.

**Tonda Gentile Romana**								
**T (°C)**	**T_2,a_ (ms)**	**A_a_ (%)**	**T_2,b_ (ms)**	**A_b_ (%)**	**T_2,c_ (ms)**	**A_c_ (%)**	**T_2,d_ (ms)**	**A_d_ (%)**
15	186 ± 3 ^f^	37.7 ± 0.1 ^d^	68 ± 1 ^f^	49.1 ± 0.5 ^a^	22.4 ± 0.7 ^a^	7.5 ± 0.3 ^c^	0.25 ± 0.01 ^c^	5.7 ± 0.1 ^d^
30	234 ± 6 ^e^	49.0 ± 0.2 ^a^	86 ± 2 ^e^	41.2 ± 0.1 ^f^	22.0 ± 2.0 ^a^	2.6 ± 0.2 ^d^	0.34 ± 0.01 ^a^	7.2 ± 0.1 ^c^
35	264 ± 8 ^d^	43.3 ± 0.1 ^c^	97 ± 2 ^d^	46.2 ± 0.0 ^c^	21.0 ± 1.0 ^a^	2.9 ± 0.1 ^d^	0.30 ± 0.01 ^b^	7.6 ± 0.1 ^b^
40	297 ± 9 ^c^	47.5 ± 0.1 ^b^	109 ± 3 ^c^	42.8 ± 0.1 ^e^	18.0 ± 1.0 ^b^	1.7 ± 0.1 ^e^	0.35 ± 0.01 ^a^	7.9 ± 0.1 ^a^
45	460 ± 10 ^b^	33.8 ± 0.2 ^e^	166 ± 3 ^b^	47.7 ± 0.1 ^b^	21.7 ± 0.2 ^a^	11.4 ± 0.3 ^a^	0.20 ± 0.01 ^d^	7.1 ± 0.1 ^c^
55	510 ± 9 ^a^	37.4 ± 0.1 ^d^	187 ± 4 ^a^	45.2 ± 0.1 ^d^	19.7 ± 0.2 ^ab^	9.4 ± 0.2 ^b^	0.28 ± 0.01 ^b^	8.1 ± 0.0 ^a^
**Tonda di Giffoni**								
**T (°C)**	**T_2,a_ (ms)**	**A_a_ (%)**	**T_2,b_ (ms)**	**A_b_ (%)**	**T_2,c_ (ms)**	**A_c_ (%)**	**T_2,d_ (ms)**	**A_d_ (%)**
8	189 ± 4 ^e^	41.7 ± 0.0 ^a^	68 ± 2 ^e^	47.0 ± 0.5 ^c^	20.0 ± 2.0 ^b^	3.7 ± 0.4 ^f^	0.21 ± 0.01 ^ab^	7.7 ± 0.1 ^b^
17	270 ± 4 ^d^	25.1 ± 0.3 ^f^	98 ± 1 ^d^	48.0 ± 0.1 ^b^	23.8 ± 0.2 ^a^	22.0 ± 0.1 ^a^	0.14 ± 0.02 ^cd^	4.9 ± 0.5 ^d^
29	362 ± 9 ^c^	26.3 ± 0.2 ^e^	128 ± 2 ^c^	49.1 ± 0.4 ^a^	22.4 ± 0.2 ^a^	18.5 ± 0.4 ^b^	0.12 ± 0.02 ^d^	6.2 ± 0.6 ^c^
41	439 ± 8 ^b^	31.8 ± 0.3 ^d^	158 ± 3 ^b^	48.5 ± 0.3 ^ab^	22.0 ± 0.3 ^a^	13.2 ± 0.4 ^c^	0.17 ± 0.02 ^c^	6.5 ± 0.4 ^c^
45	448 ± 9 ^b^	34.8 ± 0.2 ^c^	162 ± 3 ^b^	46.4 ± 0.2 ^c^	20.8 ± 0.3 ^b^	10.9 ± 0.3 ^d^	0.19 ± 0.01 ^bc^	7.8 ± 0.2 ^b^
52	550 ± 10 ^a^	35.6 ± 0.3 ^b^	192 ± 4 ^a^	46.9 ± 0.1 ^c^	19.4 ± 0.3 ^b^	8.8 ± 0.2 ^e^	0.24 ± 0.01 ^a^	8.7 ± 0.0 ^a^

Values represent mean ± standard deviation of four measurements. For each column, the different letters reveal significant differences (*p* < 0.05) according to one-way ANOVA (n = 4).

## Data Availability

The authors declare that the data supporting the findings of this study are available within the article. All the other data are available on reasonable request from the corresponding authors.
